# An In Vitro Phytohormone Survey Reveals Concerted Regulation of the Cannabis Glandular Trichome Disc Cell Proteome

**DOI:** 10.3390/plants14050694

**Published:** 2025-02-24

**Authors:** Nicolas Dimopoulos, Qi Guo, Lei Liu, Matthew Nolan, Rekhamani Das, Lennard Garcia-de Heer, Jos C. Mieog, Bronwyn J. Barkla, Tobias Kretzschmar

**Affiliations:** Faculty of Science and Engineering, Southern Cross University, Lismore, NSW 2480, Australia; nicolas.dimopoulos@scu.edu.au (N.D.);

**Keywords:** *Cannabis sativa*, jasmonic acid, salicylic acid, cytokinin, diethyldithiocarbamate, cannabinoids, leucoplast, plastid, cuticular wax, metabolism

## Abstract

Cannabis (*Cannabis sativa* L.) flower glandular trichomes (GTs) are the main site of cannabinoid synthesis. Phytohormones, such as jasmonic acid (JA) and salicylic acid (SA), have been shown to increase cannabinoid content in cannabis flowers, but how this is regulated remains unknown. This study aimed to understand which biological processes in GT disc cells phytohormones control by using an *in vitro* assay. Live GT disc cells were isolated from a high-tetrahydrocannabinol cannabis cultivar and incubated on basal media plates supplemented with either kinetin (KIN), JA, SA, abscisic acid, ethephon, gibberellic acid, brassinolide, or sodium diethyldithiocarbamate. Quantitative proteomic analysis revealed that KIN, JA, and SA caused the greatest number of changes in the GT disc cell proteome. Surprisingly, none of the treatments concertedly increased cannabinoid content or the abundance of related biosynthetic proteins in the GT, suggesting that cannabinoid increases in previous *in planta* phytohormone studies are likely due to other processes, such as increased GT density. As well, KIN-, JA-, and SA-treated GTs had numerous differentially abundant proteins in common. Several were key proteins for leucoplast differentiation, cuticular wax and fatty acid metabolism, and primary metabolism regulation, denoting that cytokinin, JA, and SA signalling are likely important for coordinating cannabis GT differentiation and development.

## 1. Introduction

Female flowers of cannabis (*Cannabis sativa* L.) possess a proliferous number of ‘peltate’ stalked glandular trichomes (GTs) on their surface [1,2,3]. These defensive structures develop predominantly in conjunction with female floral tissue and provide protection [4,5,6] by producing and storing large amounts of secondary metabolites, which include cannabinoids [1,7]. These metabolites are particularly interesting due to their medicinal properties, acting via the human endocannabinoid and central nervous systems [8,9].

Cannabis GTs have a basic morphology composed of a base that attaches to the floral epidermis, a multicellular stalk that extends outwards to act as conduit for photoassimilate transport and a globular head that is supported by the stalk [2,10]. The GT head is composed of a ring of non-photosynthetic secretory disc cells that synthesise secondary metabolites that are then stored in an extracellular storage cavity that sits adaxially to the disc cells [11]. Attaching the disc to the stalk is a cluster of photosynthetic stipe cells that sit at the centre of the disc cell ring. The cannabinoid biosynthetic pathway is highly active in the disc cells [10] and is fed by the polyketide and methylerythritol 4-phosphate (MEP) pathways that provide olivetolic acid and geranyl pyrophosphate (GPP), respectively, for the synthesis of cannabigerolic acid (CBGA), the first cannabinoid synthesised in the pathway [12]. The fatty acid hexanoic acid is the initial precursor of the cytosolically located polyketide pathway that is used to synthesise OA by way of hexanoyl-CoA synthetase, tetraketide synthase (TKS), and finally olivetolic acid cyclase (OAC) [13,14]. CBGA is then synthesised in leucoplasts by aromatic prenyltransferases, one of which being aromatic prenyltransferase 1 (CsPT1) [12], and is then transported to the storage cavity where outer cell wall-bound [11] tetrahydrocannabinolic acid synthases (THCAS) synthesise THCA from CBGA [15]. CBGA is also a precursor for other cannabinoids [16,17,18] such as cannabidiolic acid (CBDA) and cannabichromenic acid (CBCA). ‘Acid-form’ cannabinoids can be converted into their ‘neutral-form’ by auto-decarboxylation [1]. In addition, cannabis is a genetically diverse species with a wide range of chemovars [19,20], with differing GT sizes between highly cannabinoid productive modern cultivars and lower productive traditional landraces [5].

Regulation of arabidopsis (*Arabidopsis thaliana*) unicellular trichome development is well characterised, wherein the heterotrimeric MYB/bHLH/WD40 (MBW) complex acts as an activator/suppressor of GLABRA2 (GL2) to initiate trichome formation [21]. The MBW complex and GL2 act as central regulators for trichome development as they integrate together multiple phytohormonal signalling pathways, including jasmonic acid (JA), salicylic acid (SA), gibberellic acid (GA), ethylene (ETH), and cytokinins [22,23]. In contrast, regulation of multicellular trichome development is not as well understood, but recent advances have shown that the same regulatory nodes are likely broadly conserved between distantly related species [24].

Amongst phytohormones of interest, JA is often a focus of study, having been shown to stimulate both glandular and non-glandular trichome initiation in artemisia (*Artemisia annua* L.) [25], arabidopsis [26], and tomato (*Solanum lycopersicum* L.) [27]. Moreover, JA was recently demonstrated to induce increased floral cannabinoid content [28] and trichome density [29] in cannabis. Other phytohormones have been studied for their effects on trichomes. One study found that application of either auxins, GA, or JA uniformly increased non-glandular trichome density on arabidopsis and poplar (*Populus trichocarpa*), but did not increase the amount of all trichome types on tomato [23]. In another study, brassinosteroids caused tomato glandular trichome density to decrease by inhibiting JA signalling [30].

Most of these studies have focused on trichome initiation and morphogenesis, leaving open the question as to what roles phytohormones have in regulating mature trichomes, specifically in relation to their productivity. For cannabis, the question of productivity is an important one since GTs are the site for cannabinoid production, which in elite drug-type cultivars can account up to at least 20% of dry flower weight [31]. GTs in general have restructured metabolism to support high productivity; for example, tomato type VI GTs have modified central carbohydrate metabolism to feed precursors directly into secondary metabolism [32], and this is similar in cannabis [10]. Recently, productivity in cannabis GTs has been shown to be dynamic as specific cannabinoids increased with daytime progression [33], with glycolysis being an important control point. In fact, cannabinoid production can be influenced by changes in environmental growing conditions such as drought stress [34], nutrient availability [35], and photoperiod [36]. However, the main mechanisms that control GT productivity via primary metabolism [37] and the role of phytohormones are largely unknown.

Several studies have examined phytohormone regulation in cannabis, typically looking at the effects of exogenously applied compounds on floral cannabinoid content. These *in planta* experiments have found several phytohormones to be effective at changing cannabinoid production. Methyl jasmonate [28,38], salicylic acid [39], and abscisic acid [40] were found to increase cannabinoid content. Gibberellic acid decreased cannabinoids [41], while auxin analogue 1-naphthalenaecetic acid cytokinin analogue 6-benzylaminopurine [42] and ethylene donor ethephon [43] did not cause any changes. These previous studies did not discriminate between phytohormonal effects on floral tissues and trichomes. While they found cannabinoid phenotypes, the location of phytohormonal action could not be investigated in detail using *in planta* spraying. In fact, no study to date has looked at phytohormonal regulation of trichomes in isolation. Furthermore, it remains unknown if these phytohormones reach the GT disc cells and whether they would have a direct effect on GT regulation. The effects on productivity may be indirect as a result of manipulating whole plant regulatory mechanisms.

GTs are inherently challenging to study in isolation due to their microscopic size and complexity to purify in sufficient quantities. The biggest bottleneck to studying these cells is that without the ability to isolate them, it is difficult to understand the direct impact of regulatory signals on GT function. Biochemical experiments using isolated GTs have been reported for peppermint (*Mentha x piperita* L.) [44,45]. Utilising a similar system for cannabis, we studied the effects of different classes of phytohormones *in vitro* on live GT disc cells, using quantitative proteomics and targeted metabolite analysis.

We asked which phytohormones exert influence on trichome biological processes at a disc cell level in the following terms: magnitude of change in the disc cell proteome, if affected proteins have roles in trichome functioning/development, and if the cannabinoid biosynthetic pathway and cannabinoid content are affected. Since GTs are primarily defensive structures [6], we hypothesised that phytohormones involved in environmental stress response (specifically jasmonic acid, salicylic acid, and abscisic acid) will play important roles in regulating GTs and therefore may upregulate the cannabinoid biosynthetic pathway.

## 2. Results

### 2.1. Isolated Glandular Trichome Disc Cell Response to In Vitro Conditions for 24 h

A total of 1400 proteins were quantified via SWATH-MS analysis in glandular trichome disc cells across all timepoints and under all treatment conditions in the *in vitro* agar plate experimental set up (Supplementary Dataset S1).

**Figure 1:**
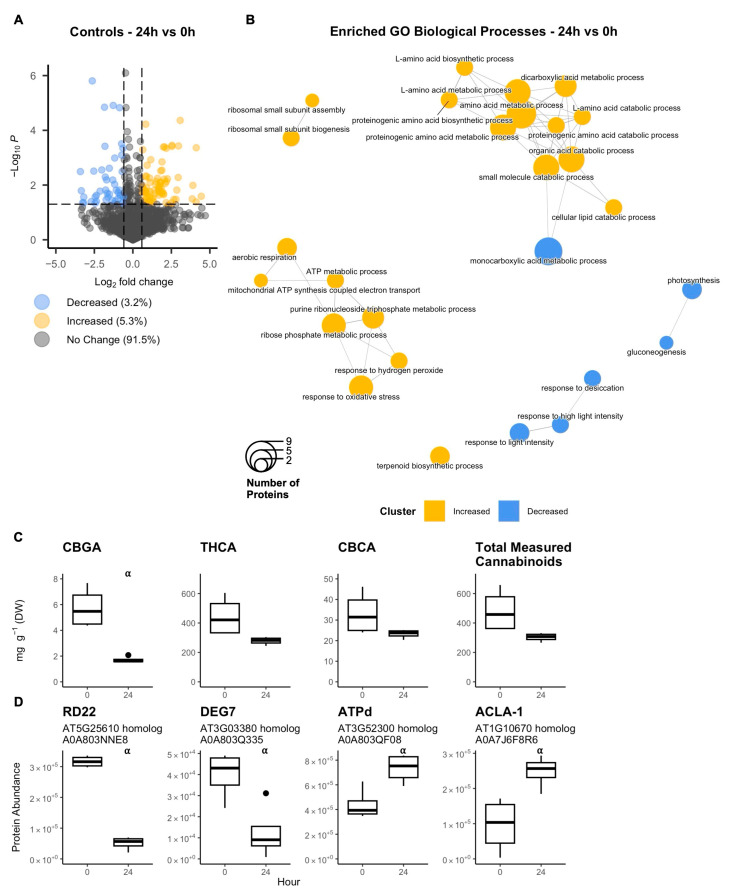
Cannabis (*Cannabis sativa* L.) glandular trichome disc cells were incubated *in vitro* on plates (*n* = 4) under control conditions over 24 h. Proteome was compared at 24 h versus 0 h (**A**), and a gene ontology (GO) enrichment analysis identified top biological processes enriched in amongst differentially abundant proteins (**B**). Trichome cannabinoid content (**C**) and abundance of key regulatory proteins were quantified (**D**). Significance (denoted by ⍺) by two-sample Student’s *t*-test. Proteins were deemed differentially abundant when *p*-value < 0.05 and when fold-change <0.67 or >1.5. Cannabinoids were significantly different if *p*-value < 0.05. Boxplots represent median, interquartile range, maximum and minimum, and outliers (closed circles). Abbreviations: CBGA, cannabigerolic acid; THCA, tetrahydrocannabinolic acid; CBCA, cannabichromenic acid; RD22, responsive to dehydration22; DEG7, degp protease 7; ATPd, mitochondrial ATP synthase subunit d; ACLA-1, ATP citrate lyase A-1. Listed in titles are protein names, closest arabidopsis homolog, and UniProt accession number.

Under control conditions, 24 h plate incubation resulted in changes to the GT disc cell proteome, with 45 decreasing differentially abundant proteins (DAPs) (3.2% of total quantified proteins) and 74 increasing DAPs (5.3% of total quantified proteins) (Figure 1A). Enrichment analysis of these DAPs identified several biological processes that were affected over the course of 24 h (Figure 1B). Biological processes (BP) that were enriched in DAPs that increased included purine metabolism, amino acid metabolism, ribosomal assembly, aerobic respiration, catabolism, and terpenoid biosynthesis. In contrast, BP enriched among decreasing DAPs included responses to desiccation and high light intensity, gluconeogenesis, and monocarboxylic acid metabolism.

Of the cannabinoids that can be quantified with the HPLC method used, only CBGA, THCA, and CBCA were measured above detectable threshold levels from all isolated GT disc cell samples.

Metabolite analysis on the isolated disc cells revealed that CBGA significantly decreased by 70% over the course of 24 h under control conditions. While not significant (*p* = 0.089), THCA also trended to decrease by 37% in content over the same period (Figure 1C).

Several key proteins involved in stress response and energy production/allocation were differentially abundant after 24 h of incubation (Figure 1D). RESPONSIVE TO DEHYDRATION22 (RD22) and DEGP PROTEASE 7 (DEG7) decreased in abundance while MITOCHODRIAL ATP SYNTHASE subunit D (ATPd) and ATP CITRATE LYASE A-1 (ACLA-1) increased.

### 2.2. Proteome Responses of Glandular Trichome Disc Cells to Phytohormone Treatments

**Figure 2:**
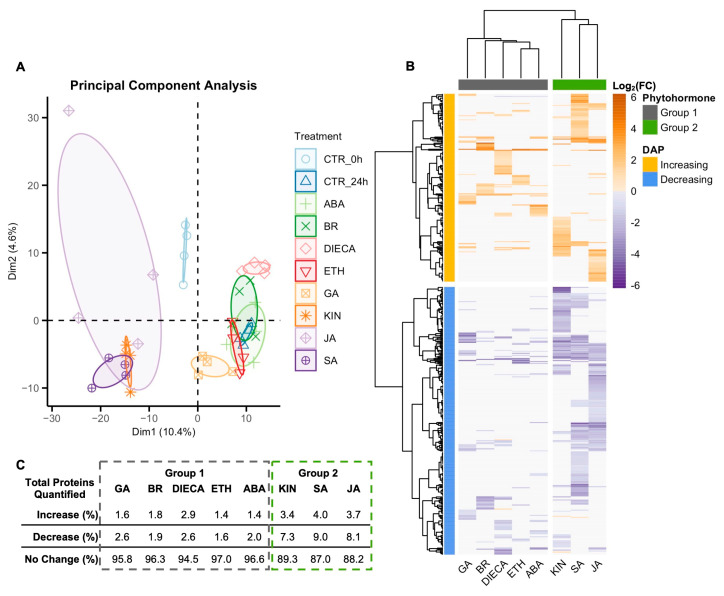
Cannabis (*Cannabis sativa* L.) glandular trichome disc cells were incubated *in vitro* on plates (*n* = 4) for 24 h with different phytohormone treatments, and their proteome was quantified (1400 proteins). Treatments were separated by principal component analysis (**A**) and hierarchical clustering (Ward’s method) by log_2_(fold-change) of the differentially abundant proteins (DAPs) (**B**). Non-significance assigned a log_2_(FC) of 0. The percent change of the total quantified proteome was tabulated for each phytohormone treatment **(C**). DAP was significant if *p* < 0.05 and fold-change either <0.67 or >1.5. Abbreviations: CTR_0h, 0 h control; CTR_24h, 24 h control; ABA, abscisic acid; BR, brassinolide; DIECA, diethyldithiocarbamate; ETH, ethephon; GA, gibberellic acid; KIN, kinetin; JA, jasmonic acid; SA, salicylic acid.

Principal component analysis (Figure 2A) revealed that the GT disc cell proteome responded differently to a range of phytohormone treatments. Treatment with ethephon (ETH), abscisic acid (ABA), or brassinolide (BR) overlapped with the ellipse from the 24 h control proteome (CTR_24h), while the response of those treated with gibberellic acid (GA), diethyldithiocarbamate (DIECA), kinetin (KIN), jasmonic acid (JA), and salicylic acid (SA) segregated away from CTR_24h. Of note, KIN, JA, and SA separated furthest away from CTR_24h. DIECA is an inhibitor of jasmonic acid synthesis, thereby inhibiting its signalling pathway [46,47].

A total of 448 proteins (32% of quantified proteome) were differentially abundant due to at least one phytohormone treatment when compared to the CTR_24h (Figure 2B). Hierarchical clustering revealed that DAPs grouped into two clusters, those that generally increased in response to a phytohormone and those that generally decreased (Figure 2B). Phytohormones grouped into two clusters as well with group 1 comprising GA, BR, DIECA, ETH, and ABA and group 2 comprising KIN, SA, and JA. DAPs in the increasing cluster were generally enriched for processes involved in carbohydrate metabolism, phenylpropanoid biosynthesis, and jasmonic acid biosynthesis, while DAPs in the decreasing cluster were enriched for catabolism, fatty acid, amino acid, and isoprenoid metabolism (Supplementary Figure S1).

Group 2 phytohormones caused the greatest change in the GT disc cell proteome (Figure 2C), with KIN, SA, and JA causing 3.4%, 4.0%, and 3.7% of the quantified proteins to increase in abundance, respectively, and 7.3%, 9.0%, and 8.1% of the proteins to decrease in abundance, respectively. Group 1 phytohormones caused a lower amount of change in the proteome with GA, BR, DIECA, ETH, and ABA causing at most 2.9% of the quantified proteins to increase in abundance and at most 2.6% to decrease.

**Figure 3:**
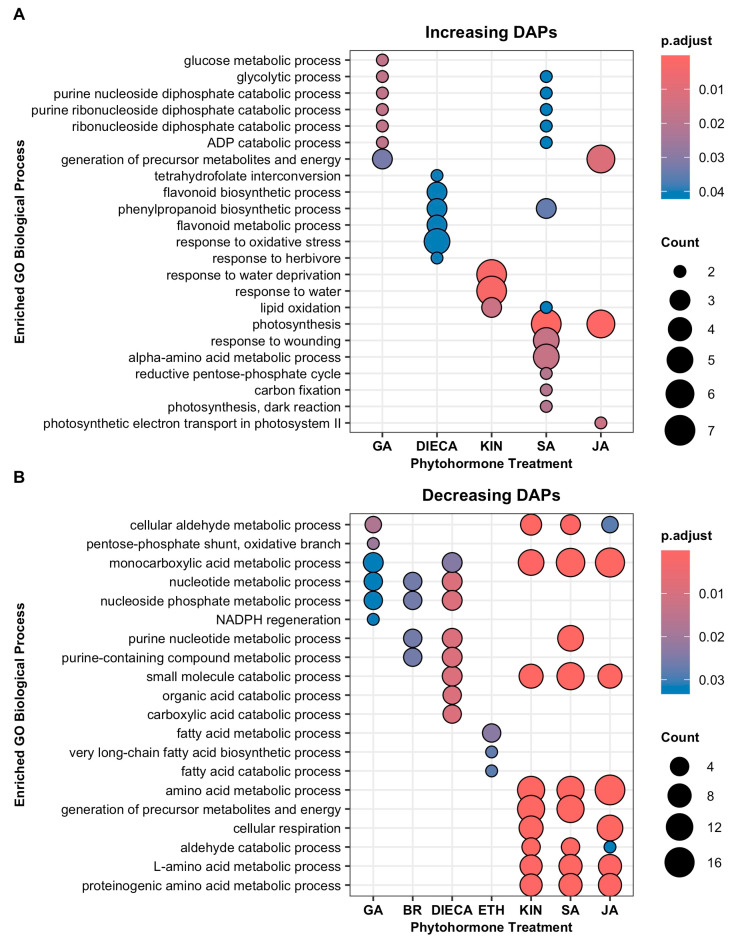
Gene ontology (GO) enrichment analysis identified enriched biological processes of increasing (**A**) and decreasing (**B**) differentially abundant proteins (DAPs) when cannabis (*Cannabis sativa* L.) glandular trichome disc cells responded to phytohormones *in vitro*. Groups that did not enrich for any processes are not shown; no enrichment was found for abscisic acid. A GO term was deemed enriched when *p*.adjust < 0.05. Count represents number of DAPs associated with GO term. Abbreviations: BR, brassinolide; DIECA, diethyldithiocarbamate; ETH, ethephon; GA, gibberellic acid; KIN, kinetin; JA, jasmonic acid; SA, salicylic acid.

Gene ontology (GO) enrichment analysis was conducted on each of the individual phytohormone DAP groups (Figure 3). For group 1 phytohormones, increasing DAPs of BR, ETH, and ABA did not cause enrichment significantly for any biological processes. DIECA caused an increased enrichment for terms involved in phenylpropanoid biosynthetic process, and the tetrahydrofolate metabolic process and GA did so for terms related to glycolysis and nucleoside catabolism (Figure 3A). In contrast, increasing DAPs in all three group 2 phytohormones caused enrichment for specific biological processes. Both SA and JA were enriched for photosynthesis and generation of precursor metabolites and energy. SA was additionally enriched for the phenylpropanoid biosynthetic process and alpha-amino acid metabolic process. KIN was only enriched for lipid oxidation and water stress (Figure 3A).

Decreasing DAPs due to the phytohormone treatments caused enrichment for a range of biological processes in GT disc cells (Figure 3B). DAPs of group 1 phytohormones were enriched for different processes with some overlap, with the exception of ABA, which was not enriched for any biological processes. Decreasing DAPs of GA, BR, and DIECA caused enrichment for nucleotide metabolic processes. DIECA additionally caused a decrease in DAPs enriched for small-molecule catabolic processes. DAPs decreased by ETH were enriched for very-long-chain fatty acid metabolism and involved the decreased abundance of eceriferum 2 (CER2) and cytochrome P450 CYP86B1 (Supplementary Figure S2). In contrast, DAPs decreased by KIN, SA, or JA were all enriched for similar biological processes. These included monocarboxylic acid metabolism, the small molecule catabolic process, cellular respiration, the generation of precursor metabolites and energy, and amino acid metabolism (Figure 3B).

**Figure 4:**
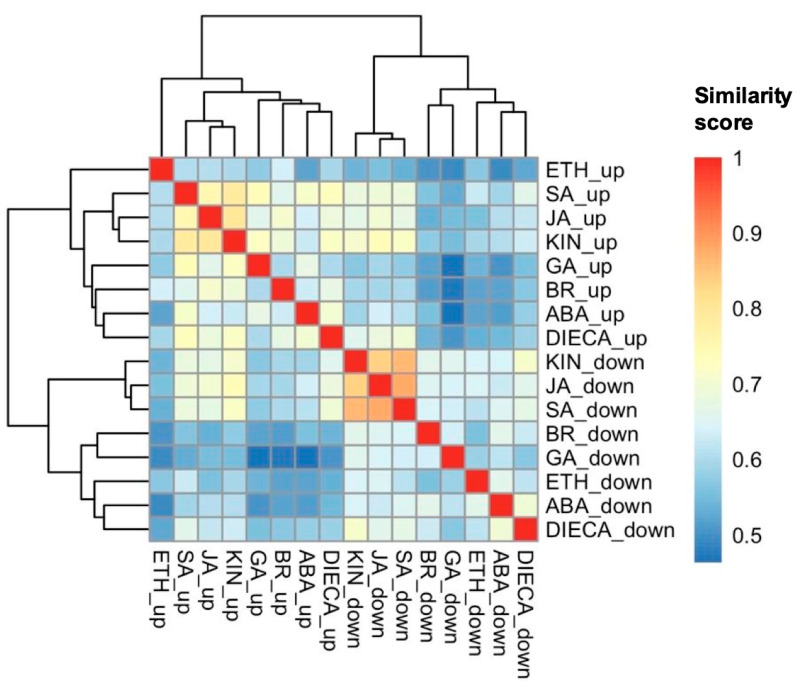
Hierarchically clustered (Ward’s method) gene cluster semantic similarity analysis of increasing (up) and decreasing (down) differentially abundant proteins when cannabis (*Cannabis sativa* L.) glandular trichome disc cells responded to phytohormones *in vitro*. Similarity analysis used the gene ontology of the closest arabidopsis homologs of the cannabis proteins of interest. Abbreviations: ABA, abscisic acid; BR, brassinolide; DIECA, diethyldithiocarbamate; ETH, ethephon; GA, gibberellic acid; KIN, kinetin; JA, jasmonic acid; SAL, salicylic acid.

Gene ontology gene cluster semantic similarity allowed for the pairwise comparison between each group of decreasing and increasing DAPs resulting from the phytohormone treatments in order to measure how biologically similar they are to one another (Figure 4). Hierarchically clustering the similarity scores identified that the groups of decreasing DAPs due to KIN, SA, or JA treatments had high biological similarity to each other. This was also the case for the groups of increasing DAPs due to KIN, SA, or JA.

### 2.3. DAPs and Biological Processes Shared Between Group 2 Phytohormones

**Figure 5:**
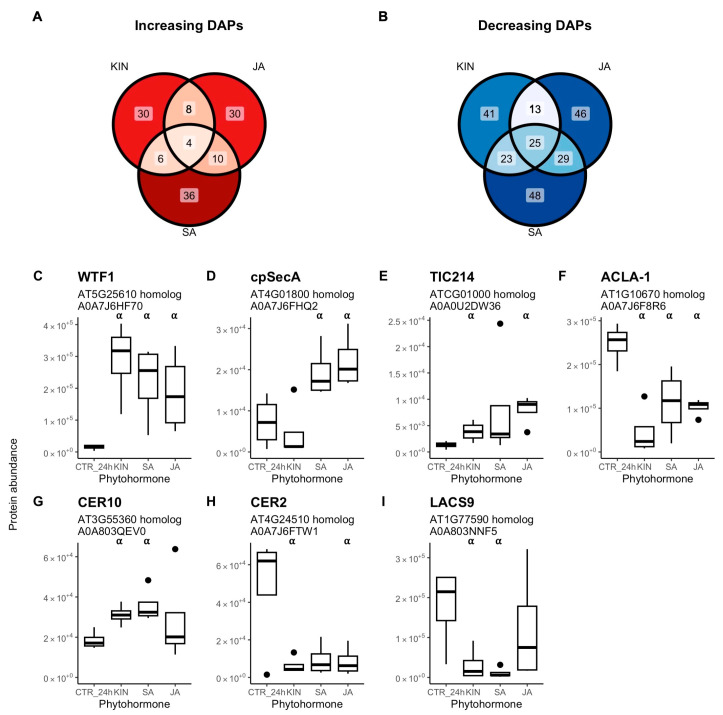
Comparison of differentially abundant proteins (DAPs) in cannabis (*Cannabis sativa* L.) glandular trichome disc cells when treated *in vitro* (*n* = 4) with either kinetin (KIN), jasmonic acid (JA), or salicylic acid (SA). Venn diagrams represent intersecting increasing (**A**) and decreasing (**B**) differentially DAPs between KIN, JA, and SA. Key differentially abundant proteins affected by at least two of the phytohormones included those related to plastidial function (**C**–**E**), cytosolic acetyl-CoA synthesis (**F**), and fatty acid metabolism (**G**–**I**). Boxplots represent median, interquartile range, maximum, minimum, and outliers (closed circles). Significance (denoted by ⍺) determined by two-sample Student’s *t*-test (*p*-value < 0.05) by comparing phytohormone treatments (*n* = 4) to the control (CTR_24h). Abbreviations: WTF1, WHAT’S THIS FACTOR 1; cpSecA, chloroplastic protein translocase subunit SECA1; TIC214, translocon at the inner envelope membrane of chloroplasts 214; ACLA-1, ATP citrate lyase A-1; CER10, very-long-chain enoyl-CoA reductase; CER2, eceriferum 2; LACS9, chloroplastic long chain acyl-CoA synthetase 9. Listed in titles are protein names, closest arabidopsis homolog, and UniProt accession number.

Amongst group 2 phytohormones, 126 unique DAPs increased, of which 28 (22%) were affected by at least two of three phytohormones of the group (Figure 5A). Similarly, 225 unique DAPs decreased, with 90 (40%) of them affected by at least two of three phytohormones (Figure 5B).

Seven DAPs of interest were identified that exclusively increased or decreased by at least two of the following phytohormones: KIN, SA, and/or JA. These are implicated in biological processes involving trichome differentiation/morphogenesis, carbohydrate metabolism, or fatty acid metabolism. Three DAPs involved in plastidial function (Figure 5C–E) included WHAT’S THIS FACTOR 1 (WTF1) that was increased by KIN, SA, and JA; chloroplastic protein translocase subunit SECA1 (cpSecA) that was increased by SA and JA; and translocon at the inner envelope membrane of chloroplasts 214 (TIC214) that was increased by KIN and JA. ACLA-1 decreased in the presence of KIN, SA, and JA, and it is involved in cytosolic acetyl-CoA synthesis (Figure 5F). Lastly, three DAPS involved in fatty acid metabolism (Figure 5G–I) were affected. These included very-long-chain enoyl-CoA reductase (CER10), which increased due to KIN and SA; eceriferum 2 (CER2), which decreased due to KIN and JA; and chloroplastic long chain acyl-CoA synthetase 9 (LACS9), which decreased due to KIN and SA.

### 2.4. Phytohormonal Effects on Cannabinoid Metabolism

**Figure 6:**
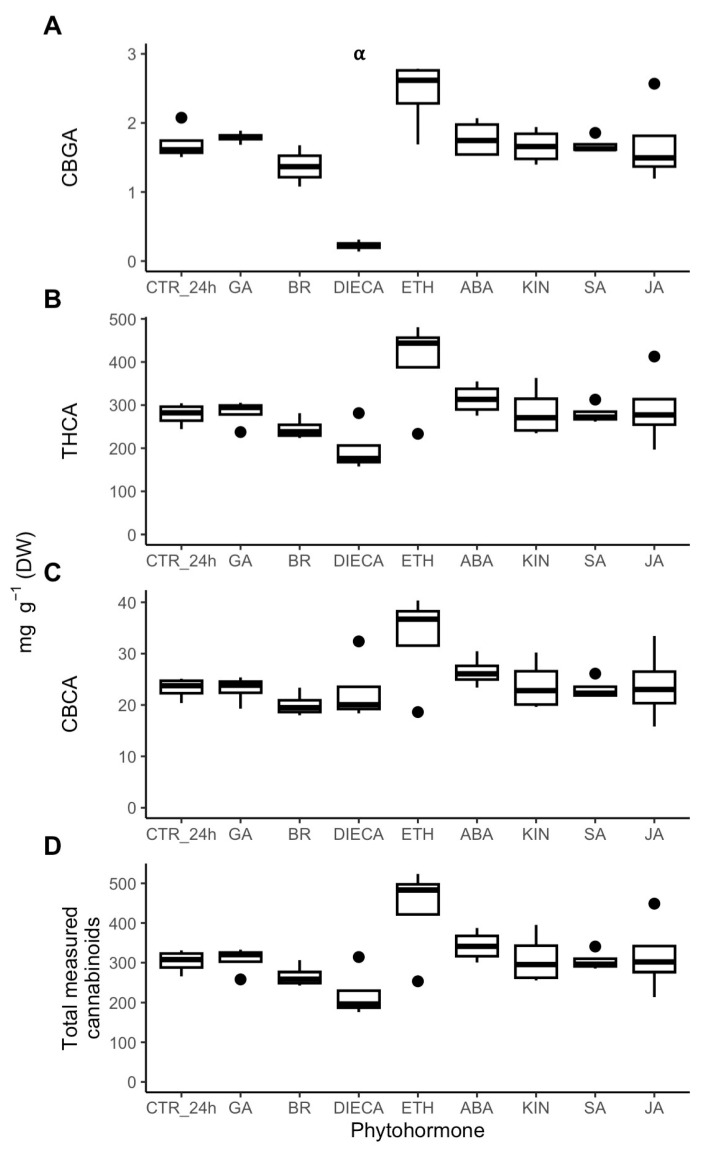
CBGA (**A**), THCA (**B**), CBCA (**C**), and total measured cannabinoid (**D**) content [mg g^−1^ (DW)] of isolated cannabis (*Cannabis sativa* L.) glandular trichome disc cells when treated *in vitro* with phytohormones (*n* = 4). Boxplots represent median, interquartile range, maximum and minimum, and outliers (closed circles). Significance (denoted by ⍺) was determined by two-sample Student’s *t*-test (*p*-value < 0.05) by comparing phytohormone treatments to the control (CTR_24h). Abbreviations: CBGA, cannabigerolic acid; THCA, tetrahydrocannabinolic acid; CBCA, cannabichromenic acid; ABA, abscisic acid; BR, brassinolide; DIECA, diethyldithiocarbamate; ETH, ethephon; GA, gibberellic acid; KIN, kinetin; JA, jasmonic acid; SA, salicylic acid.

Phytohormonal effects on disc cell cannabinoid content were measured and compared to the CTR_24h values (Figure 6). DIECA application resulted in a significant 87% decrease in CBGA, and while not significant (*p* = 0.058), THCA trended to decrease by 29%. ETH also caused a notable trend (*p* = 0.058) by increasing CBGA by 43%. CBCA and total measured cannabinoid content did not change for any of the treatments.

**Figure 7:**
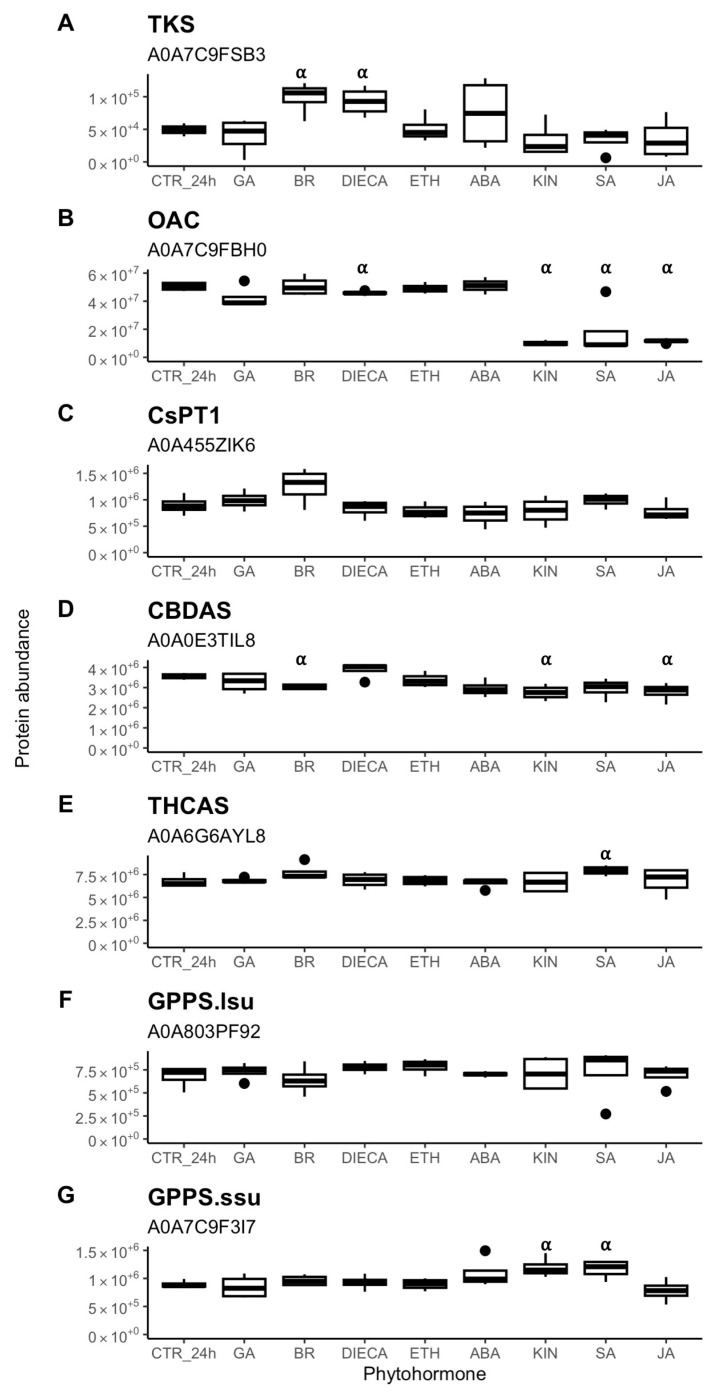
Abundance of key proteins involved in cannabinoid biosynthesis (**A**–**E**) and geranyl pyrophosphate synthesis (**F**,**G**) in cannabis (*Cannabis sativa* L.) glandular trichome disc cells when treated *in vitro* with phytohormones (*n* = 4). Boxplots represent median, interquartile range, maximum, minimum, and outliers (closed circles). Significance (denoted by ⍺) was determined by two-sample Student’s *t*-test (*p*-value < 0.05) by comparing phytohormone treatments to the control (CTR_24h). Abbreviations: ABA, abscisic acid; BR, brassinolide; DIECA, diethyldithiocarbamate; ETH, ethephon; GA, gibberellic acid; KIN, kinetin; JA, jasmonic acid; SA, salicylic acid; TKS, tetraketide synthase; OAC, olivetolic acid synthase; CsPT1, *Cannabis sativa* aromatic prenyltransferase 1; CBDAS, cannabidiolic acid synthase; THCAS, tetrahydrocannabinolic acid synthase; GPPS.lsu, geranylgeranyl pyrophosphate synthase 1 GPPLsu; GPPS.ssu, geranylgeranyl pyrophosphate reductase GPPSsu. UniProt accession number listed underneath protein names.

SWATH-MS allowed the relative abundance levels of seven key cannabinoid biosynthetic proteins (Figure 7) to be calculated: tetraketide synthase (TKS), olivetolic acid cyclase (OAC), aromatic prenyltransferase1 (CsPT1), cannabidiolic acid synthase (CBDAS), tetrahydrocannabinolic acid synthase (THCAS), geranylgeranyl pyrophosphate synthase 1 GPPLsu (GPPS.lsu), and geranylgeranyl pyrophosphate reductase GPPSsu (GPPS.ssu). Neither GA, ETH, nor ABA had any effect on the abundance of these seven proteins. Both BR and DIECA increased TKS abundance by 100% and 88%, respectively. DIECA, KIN, SA, and JA decreased OAC abundance by 9%, 80%, 77%, and 64%, respectively. BR, KIN, and JA decreased CBDAS abundance by 15%, 23%, and 22%, respectively. SA increased THCAS abundance by 17%. KIN and SA increased GPPS.ssu abundance by 34% and 31%, respectively. None of the treatments had any effect on the abundance of CsPT1 or GPPS.lsu.

## 3. Discussion

### 3.1. In Vitro GT Assay Proof of Concept

*In vitro* experiments have previously been carried out on isolated peppermint GTs to study how carbon flux in these cells affect terpenoid biosynthesis; these experiments used small amounts of trichome heads which were incubated in a liquid culture for only 1 h [44,45]. In this study, we wanted to understand phytohormonal control of cannabis GTs in *in vitro* isolated conditions to remove the potential effects of other signalling pathways that would be active in *in planta* conditions.

Unlike McCaskill et al. [45], solid plate culturing was chosen since initial attempts of culturing in liquid media proved unsuccessful. Cannabis GT cells hyperaccumulated polyphenolic pigments when in liquid, indicating a high level of cell stress. We surmised that this was due to the relatively anoxic environment of the liquid media compared to the solid plate culture. The GT disc cells were incubated for 24 h to give enough time for turnover of the proteome and changes in CBGA content in response to treatments. As an intermediate cannabinoid, CBGA experiences high flux, and abundance can change in as little as 6 h. Changes in abundance can be used as a broad gauge for cannabinoid pathway activity [33]. The cells were incubated in constant light to mimic daytime conditions, which is when cannabis GTs are most metabolically active for cannabinoid biosynthesis [33].

We initially expected that GT disc cells would experience some level of stress in the *in vitro* assay, but proteomic data suggested otherwise. Separated from their stalks that are the efficient conduits supplying photoassimilates [10], the GTs were in an artificial environment where nutrient availability varies from what is supplied when attached on the plant. Some increased DAPs (Supplementary Figure S3) such as a sucrose synthase (SUS3) and several aminotransferases and synthetases (ALAAT2, ATCS-C, and ASN1) may indicate to sugar starvation [48].

Yet, changes in other key regulators indicate that the cells were not metabolically stressed. The structural subunit d of mitochondrial ATP synthase (ATPd) increased in abundance (Figure 1D), indicative of greater ATP generation [49,50]. While not enzymatic, ATPd is an essential subunit for the ATP synthase complex to be in functional conformation [51]. The subunit has a regulatory role in ATP generation [49] as higher expression directly results in a higher ATP/ADP ratio in cotton ovule fibre cells (a type of trichome) [50]. Importantly, ACLA-1 also increased in abundance (Figure 1D). By being a non-redundant subunit of ATP citrate lyase (ACL), ACLA-1 acts as a regulator of ACL activity and controls the available pool of cytosolic acetyl-CoA in the cells and how resources are allocated to different metabolic pathways [52,53,54]. A decrease in ACLA-1 in arabidopsis induces metabolic stress and upregulation of stress response genes, causing the plant to reallocate the limited pool of acetyl-CoA to specific metabolic pathways, such as anthocyanin biosynthesis [53]. Overexpression of ACLA-1 in arabidopsis leads to elevated ACL activity, producing a greater pool of acetyl-CoA and a different shift in resource allocation whereby cutin and cuticular wax biosynthesis is upregulated rather than other lipid metabolites like glycerolipids and sterols [52]. Thus, an increase in ACLA-1 would indicate that GT disc cells were not stressed and have carbon resources available.

In addition, we observed that the cells responded accordingly to lower levels of high light stress and osmotic stress. DEG7 (Figure 1D), a plastidial DegP protease that responds to high light conditions and damage by photoinhibition [55], and RD22 (Figure 1D), a key positive regulator of drought stress response [56], both decreased in abundance. Lastly, microbial contamination was likely not a stressor for GT disc cells since none of the DAPs were enriched for biotic stress response at 24 h (Figure 1B) and microbial contamination was not apparent until 48 h in initial time course trials (Supplementary Figure S4).

The evidence suggested continued and sustained secondary metabolism by GT cells after 24 h of incubation under control conditions. Key enzymes involved in cannabinoid biosynthesis (TKS, OAC, CsPT1, THCAS, CBDAS, GPPS.ssu, GPPS.lsu) did not change in abundance (Supplementary Figure S3), indicating that the cannabinoid pathway was affected by the *in vitro* conditions. Correspondingly, THCA, CBCA, and total measured cannabinoid content did not significantly change (Figure 1C). While CBGA content did decrease after 24 h (Figure 1C), its continued presence would indicate to sustained CBGA synthesis, albeit at a lower rate. Otherwise, if CBGA synthesis had halted, then its content would have decreased to negligeable amounts, as it did with the DIECA treatment (Figure 6A). Cannabinoids are produced specifically by the non-photosynthetic secretory cells in the GT disc, making the GT disc a strong sink tissue reliant on photoassimilate import via the GT stalk [10]. This means that CBGA content likely correlates with the amount of photoassimilates available to the GT [33]. Thus, the lower CBGA content seen after 24 h would suggest that sugar availability to GTs *in vitro* was lower than normally found *in planta*, which fits the increased SUS3 abundance seen *in vitro* (Supplementary Figure S3).

### 3.2. Phytohormone Regulation of Cannabinoid Biosynthesis

The *in vitro* phytohormone treatments at large did not cause concerted changes in cannabinoid pathway biosynthetic proteins (Figure 7) and cannabinoid content (Figure 6) within a period of 24 h. This juxtaposes several *in planta* studies where phytohormones were tested by spraying on plants and changes in floral cannabinoid flower and/or gene expression were assessed. In these studies, GA [41] was found to decrease total tetrahydrocannabinol (THC) content after 24 h, while ABA [40] caused total THC content to increase 24 h after 3 days of treatment. JA [28,38,57] caused total THC content to increase after several weeks of treatment. ETH [43] caused cannabidiol to increase 24 h after 6 days of treatment. SA caused THCA [57] content to decrease 1 week after 8 weeks of treatment. Garrido et al. [57] also tested if JA or SA changed the gene expression of either *OAC*, *CBDAS*, or *THCAS* and found neither treatment had effects on these enzymes. In contrast, Sands et al. [39] reported that SA positively regulated the expression of *TKS*, *OAC*, *CsPT1*, *CBDAS*, and *GPPS.ssu* as early as three hours after treatment. The differences in gene expression results could be due to the different cultivars and/or treatment methods used. For example, Garrido et al. [57] applied weekly foliar sprays throughout floral development, while Sands et al. [39] used a onetime foliar application. These *in planta* studies were unable to explain the actual mechanisms that resulted in changed cannabinoid content in the GT since they were limited to examining at the floral tissue level. The lack of change in cannabinoid content and proteins in our *in vitro* experiment compared to the changes in the whole plant experiments point to phytohormones exerting control on cannabinoid biosynthesis indirectly *in planta*. In fact, Garrido et al. [57] found that cannabinoid content correlated with plant growth and not with expression of the biosynthetic genes. Likely avenues of phytohormonal control over cannabinoid biosynthesis include manipulating primary metabolism and resource allocation in the short term or affecting trichome density in the long term. This may explain the differences in cannabinoid composition seen between different cannabis chemovars [20] or when cannabis responds to environmental stresses [34].

### 3.3. GT Changes in Common Between Kinetin, Jasmonic Acid, and Salicylic Acid Indicate Roles in Coordinating Trichome Specific Features

Group 2, comprised of KIN, SA, and JA (Figure 2B), caused much larger proteome changes (greater than 10% each) than those observed for group 1 hormones. These phytohormones induced responses in the GT disc cells that were biologically similar to each other (Figure 4), as evidenced by a large number of increasing and decreasing DAPs in common (Figure 5). Significant interplay between these three hormones occurs in plants. JA and SA are largely antagonistic to each other to reduce redundancy [58], while cytokinins act synergistically with SA to activate pathogen defences [59], as well with JA to confer resistance to feeding insects [60]. Interestingly, we found that KIN, JA, and SA caused OAC to decrease in abundance in a similar fashion by an average fold-change of 0.26 times (Figure 7), which may point to a common regulatory mechanism of OAC shared by the three. This stands in contrast to Sands et al. [39] who had found that SA increased *OAC* expression.

The SWATH-MS analysis we employed was not sensitive enough to detect and quantify relatively low abundant regulatory proteins which include those of the MBW complex and receptors of various phytohormone signalling pathways. However, we were able to quantify homologs of the ABA receptor PYR1 [61] and BEN1, a regulator in BA signalling [62]. Neither of them changed in abundance in response to ABA or BA, respectively (Supplementary Figure S5), indicating that GT disc cells are likely relatively insensitive to these phytohormones, as seen by the small number of DAPs caused by ABA and BA (Figure 2).

In arabidopsis [21] and conserved in other species [24], trichome initiation is centrally controlled by the MBW complex where complex developmental, stress, and phytohormonal signals are integrated through it [21], which include the KIN, JA, and SA signalling pathways [22]. Together, the magnitude of impact (Figure 2) and similarity in responses (Figure 4 and Figure 5) suggest that KIN, SA, and JA may have important roles in regulating cannabis trichome development, morphology, and/or productivity by regulating many of the same biological processes (Figure 3).

When examined in detail, seven DAPs that were affected by either KIN, SA, and/or JA point to these phytohormones’ involvement in trichome differentiation and morphogenesis processes (Figure 5C–I). These included CER10, CER2, and LACS9 that are involved in very-long-chain fatty acid (VLCFA) elongation for cuticular wax synthesis, as well as cpSecA, TC214, and WTF1, which are involved in plastidial function and development. Also important was ACLA-1, which is involved in cytosolic acetyl-CoA synthesis.

CER10 is an enoyl-CoA reductase involved in VLCFA elongation that is required for cuticular wax synthesis. It has been shown to have a conserved role in trichome development in both tomato and arabidopsis [63], and knockout mutants resulted in reduced trichome densities. Cannabis GTs have prolonged development that spans flower development [2] and involves remodelling of the cell wall and cuticle thickening of the apical surface of disc cells to create the storage cavity [64]. Not only did we see CER10 increasing when mature GTs were treated with either KIN, JA, or SA (known to positively regulate trichome initiation across species), but it also decreased in the 24 h vs. 0 h controls when no exogenous hormones were present (Supplementary Figure S3). This would suggest that CER10 has a role in regulating GT differentiation that would be likely linked to cell wall synthesis and storage cavity formation. This further implies that constant rates of JA, SA, or KIN may be needed to maintain GT homeostasis.

Changes in CER2 and LACS9 abundance by KIN, SA, and JA further substantiate their roles in directing cuticular wax formation in GTs (Figure 5H–I). CER2 is required for the fatty acid elongase complex to synthesise VLCFAs of lengths greater than 28 carbons [65]. We observed that the three phytohormones caused CER2 abundance to decrease by an average of 83%, indicating that GT cuticular wax composition would be shifted, possibly to shorter-chain-length VLFCAs, by these phytohormones. This parallels what is seen in arabidopsis trichomes which have an altered cuticular wax composition and is attributable to altered expression of the cuticular wax pathway that includes low expression of *CER2* [66,67]. LACS are a family of long-chain acyl-CoA synthetases that catalyse free fatty acids into fatty acyl-CoAs and are involved in fatty acid transport in the cell [68,69]. In arabidopsis, LACS9 is responsible for 90% of LACS activity in leaf plastids [70], thus having it decrease by KIN and SA may indicate to a shift in fatty acid allocation in cannabis GT metabolism.

Increased abundances of plastidial proteins cpSecA, TIC214, and WTF1 by KIN, SA, and JA suggests roles in regulating GT leucoplast function (Figure 5C–E). CpSecA is a translocation ATPase and part of the chloroplast secretory (cpSec) pathway [71]. The cpSec pathway is responsible for the transport of unfolded proteins into the chloroplast lumen and is essential for the development and differentiation of chloroplasts and all other plastid types [72,73]. Proper functioning of this pathway is essential for trichome development, as ATcpSecA-deficient mutants result in malformed arabidopsis trichomes [71]. TIC214 is a component of the TOC-TIC system, another plastidial protein transport system, where direct regulation of TOC-TIC plays a role in proteome remodelling and plastid biogenesis [74]. WTF1 is also important for plastidial function, as it is a required component of the splicing machinery in chloroplasts that splices group II introns, and it is needed for proper chloroplast function [75]. Interestingly, WTF1 decreased in abundance in the 24 h control (Supplementary Figure S3), and only KIN, JA, and SA were able to restore abundance (Figure 5C). These changes would suggest that KIN, JA, and SA have a role in regulating leucoplast differentiation in cannabis GT non-photosynthetic secretory cells, which are sites of secondary metabolite synthesis [11].

Common decreasing DAPs were enriched for multiple components of primary metabolism, and they could indicate a role for KIN, JA, and SA in the restructuring metabolism that occurs during trichome development, where primary metabolism is streamlined to feed into cannabinoid synthesis. An indication that resource allocation in GTs is manipulated by these three phytohormones is the decrease in ACLA-1 in treated trichomes (Figure 5F). ACLA-1 is a non-redundant subunit of ATP citrate lyase, and a decrease would result in limiting the cytosolic acetyl-CoA pool which plants typically respond to by prioritising acetyl-CoA to certain metabolic processes that include stress responses [53]. Primary metabolism in photosynthetic tomato type VI trichomes is heavily modified with the uncoupling of light photosynthesis from dark reactions. While tomato trichomes receive their carbon from leaf sucrose, the uncoupling allows energy and reducing power from GT photosynthesis to directly feed into their secondary metabolism [32]. A similar mechanism is thought to exist in cannabis GTs, but in this case, it is the GT disc’s central stipe cells (these interface between the secretory cells and the stalk) that are photosynthetic and provide reducing power to the secretory cells’ secondary metabolism [10]. Several subunits of photosystems I and II also increased in abundance by KIN, JA, and SA (Supplementary Figure S6), which point to possible upregulation of photosynthesis in the stipe cells, as disc cells are non-photosynthetic, leading to possible greater production of reducing equivalents [10,32].

### 3.4. Ethephon Is a Possible Positive Regulator of Cannabinoid Biosynthesis

ETH caused a near significant (*p* = 0.058) 43% increase in CBGA content (Figure 6A), indicating that ethylene signalling may be a potential positive regulator of cannabinoid biosynthesis, which would need further testing to validate. The phytohormone did not change the abundance of any enzymes involved in cannabinoid biosynthesis (Figure 7); rather, a possible explanation would involve ETH adjusting resource allocation towards cannabinoid production. Decreasing DAPs affected by ETH enriched for very-long-chain fatty acid (VLCFA) metabolism which included the enzymes CER2 and CYP86B1 (which was only affected by ETH); in addition, ACLA-1 was unaffected by ETH (Supplementary Figure S2). CYP86B1 is a member of the cytochrome P450 CYP86 subfamily which is involved in fatty acid ω-hydroxylation required for suberin and cutin synthesis in cuticle metabolism [76,77]. In intact GTs, the cuticular layer covering the extracellular storage cavity continuously thickens as the cavity fills during GT development [64,78], indicating that cuticle metabolism is likely a major sink for resources in GTs. Hence, decreasing CYP86B1 abundance could potentially limit cuticle metabolism and reduce this pathway’s strong sink strength for acetyl-CoA [52] in the GT, thereby making more acetyl-CoA available for cannabinoid biosynthesis.

### 3.5. Diethyldithiocarbamate (DIECA) Is a Negative Regulator of Cannabinoid Biosynthesis

DIECA is a strong reducing agent that can be used to reduce 13(S)-hydroperoxylinolenic acid (HPOTrE) to 13-hydrolinolenic acid in plants, thereby reducing the pool of HPOTrE that is a precursor for cyclisation into jasmonic acid [46]. The compound can be used exogenously to disrupt JA signalling *in planta*, as done to induce powdery resistance in wheat (*Triticum aestivum*) [47]. While a changing proteome of the GT disc cells treated with DIECA indicated that they were still alive, the observed increases in phenylpropanoid and tetrahydrofolate metabolism together with the decrease in catabolism would indicate that they were likely stressed (Figure 3). Also indicative of stress is that DIECA caused a decrease in CBGA, one of the GT’s main metabolic outputs (Figure 6A), without a concomitant decrease in abundance of cannabinoid biosynthesis proteins (Figure 7).

While negative regulation of cannabinoid biosynthesis by DIECA cannot be readily explained, it is indicative that disruption of JA signalling is stressful to cannabis GTs and that JA may be needed for trichome homeostasis. One possible mechanism could be that DIECA disrupts fatty acid metabolism in the trichome, thereby limiting the availability of hexanoic acid needed for olivetolic acid biosynthesis and subsequently causing CBGA content to decrease. Fatty acid synthesis occurs in plastids, and since it appears that JA is important for proper plastid function in trichomes (Figure 5), disruption of JA signalling by DIECA could interfere with trichome leucoplast function. DIECA has been observed to affect fatty acid metabolism in other species, where it modulated fatty acid composition in wheat [47], and decreased fatty acid content in grape berries (*Vitis vinifera* L.) [79].

### 3.6. Jasmonic Acid Is a Key Coordinating Phytohormone for GT Initiation and Development in Cannabis

Our study supports recent research that established JA signalling as a key regulatory pathway for the initiation, development, and function of cannabis glandular trichomes. While foliar application of JA caused increased cannabinoid content in floral tissue [28], this increase is unlikely due to upregulation of the cannabinoid biosynthetic pathway. CBGA synthesis is a likely rate-limiting step in the pathway [33] and is synthesised by two aromatic prenyltransferases: CsPT1 and CsPT4. The expression of these two enzymes has been found to be unresponsive to methyl jasmonate, and promoter regions of their genes do not have JA-responsive elements [39]. We similarly found that JA did not affect the abundance of CsPT1, tetraketide synthase, THCA synthase, or subunits of GPP synthase (GPPS.ssu and GPPS.lsu), while it caused a decrease in olivetolic acid cyclase and CBDA synthase (Figure 7). Congruently, exposure of GT disc cells to JA did not increase either CBGA or THCA content *in vitro* (Figure 6A, B). Consequently, increased floral cannabinoid content *in planta* by JA [28] may be caused by some mechanism other than upregulation of biosynthetic enzymes. This could either be by increasing the allocation of carbon to secondary metabolism, which some plants do when under stress [80], or by promoting GT initiation to increase overall floral GT density, a phenomenon recently observed in cannabis [29].

**Figure 8:**
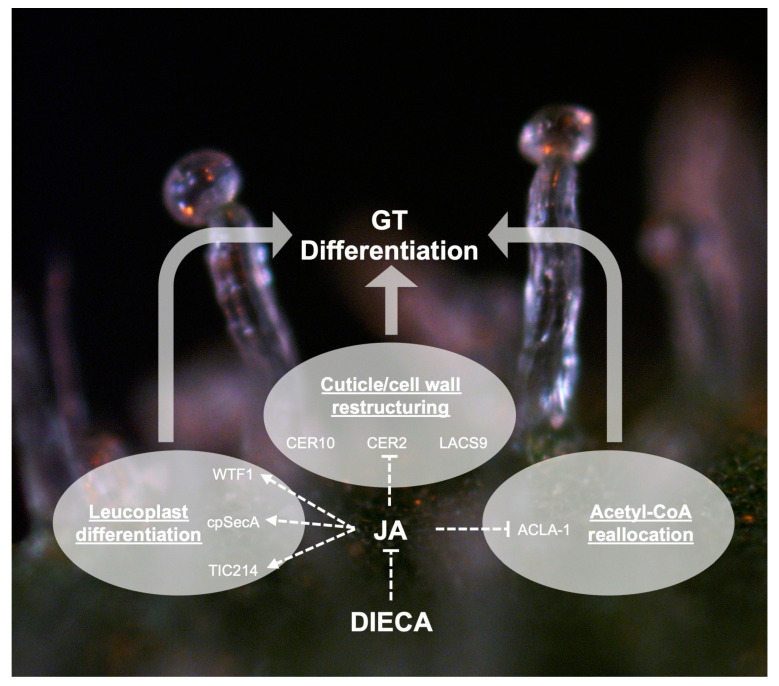
A proposed model for the control of processes important for cannabis (*Cannabis sativa* L.) glandular trichome (GT) differentiation by jasmonic acid (JA), which positively (dashed arrows) and negatively (dashed bars) regulate specific key enzymes. Diethyldithiocarbamate (DIECA) negatively affects GT differentiation by inhibiting JA signalling.

Huang et al. [29] found that a methyl jasmonate (MeJA) mediated pathway was involved in trichome formation. Foliar application of MeJA increased trichome density on cannabis flowers and RNA-Seq analysis detected a cluster of MeJA responsive genes that strongly correlated with GT formation during flower development. CsMYC4, a bHLH transcription factor (TF), was identified as a key regulatory gene in the cluster and is a close homolog of the trichome inducing SlMYC1 TF in tomato [81]. Consequently, Huang et al. [29] proposed a MYC-JAZ complex as the JA induction mechanism for GT initiation. This is similar to the mechanism in tomato and artemisia [82] and the MBW model of multicellular trichome initiation [24]. Our research further substantiates Huang et al.’s [29] findings that JA is important for cannabis GT development. We observed that 11.8% of quantified proteins responded to JA in mature GTs that were sampled at 6 weeks into flowering. As well, we found that treating GTs with DIECA likely inhibited the jasmonic acid pathway, which resulted in GTs showing signs of a stress response. These two observations indicated that mature GTs are receptive to jasmonic acid signalling past the trichome initiation stage, and that JA is likely important for maintaining GT homeostasis. Furthermore, JA regulated key proteins involved in leucoplast formation, cuticle and cell wall modification, and energy metabolism (via acetyl-CoA reallocation), which are processes needed for the development of mature cannabis GTs (Figure 8). Finally, the similarity of the KIN and SA responses to JA suggests a more complex integration of hormonal interplay to be of importance for GT homeostasis in cannabis.

## 4. Conclusions

In conclusion, we have shown that of the phytohormones tested, JA, SA, and KIN, exerted the largest effects on the cannabis GT proteome, with a significant amount of similarity in the responses between them. The results suggested a role for these three hormones in trichome initiation and development as proteins involved in plastidial differentiation, cuticle and cell wall modification, and energy metabolism changed in abundance. This study further corroborates recent findings that JA signalling positively regulates trichome development in cannabis. Finally, none of the phytohormones caused any concerted changes in cannabinoid pathway protein abundance or cannabinoid content. This would suggest that phytohormones control *in planta* cannabinoid content indirectly by manipulating resource allocation and/or trichome development.

## 5. Materials and Methods

### 5.1. Grow Conditions and Plant Propagation

The High-THC Hindu Kush cultivar of *Cannabis sativa* L. was provided by Cann Group Ltd. (Melbourne, Australia) for our study. Plants were grown in a secure grow room at Southern Cross University under an authority issued by the New South Wales Department of Health.

Clonal plants were propagated from cuttings taken from mother plants and implanted into wetted rockwool plugs with Clonex^®^ rooting gel (3.0 g/L indole acetic acid). The clones were then incubated in a growth chamber (Conviron ATC60) for 2 weeks at 28 °C, under long-day cycles (20 h light/4 h dark) and ambient relative humidity to promote root development.

Once roots were established, clones were potted into 5 L pots with potting mix (70:30% blend of coco–perlite) and osmocote fertiliser (6.4 g/L) and transferred into a large grow tent in the grow room for 26 vegetative growth days. The grow room was set at 26 °C with ambient relative humidity. Plants were exposed to long-day cycles (20 h light/4 h dark) using LED lighting (ViparSpectra©, Richmond, CA, USA) at constant intensity (800 µmol m^2^ s^−1^ at canopy height), in the absence of UV-B light. Plants were watered daily with tap water.

After 44 days, plants were moved out of the tent and onto grow room benches with a capillary watering system. Flowering was induced with short-day light cycles (12 h light/12 h dark) using LED lighting. PAR during flowering was constant throughout the day at an average of 650 µmol m^−2^ s^−1^ at the top of plant canopies, approximately 60 cm below LED lights. Plants were used for experiments at 49 flowering days (from onset of short-day cycle), where flowers developed to mid-maturity (30–50% pistils senescing, and flower buds have taken on a “frosty” look due to proliferous GT development).

### 5.2. Trichome Disc Cell Isolation

Cannabis disc cells were isolated using the same method as in Dimopoulos et al. [33]. Flowers from four plants were carefully harvested to avoid cutting the sugar leaves or bracts and to exclude fan leaves from the harvested material to ensure no mesophyll tissue contamination in the final trichome isolation. Flowers were first dipped in ice-cold ethanol (100% *v*/*v*) for 15 s and then dipped twice in ice-cold water for 20 s each to promote the rupturing and washing away of the trichome resin storage cavity.

After dipping, the batch of flowers was then gently washed in a small washing machine (BubbleBagDude©, Sherrills Ford, NC, USA; Bubble Machine) for 10 min with ice-cold water to detach the GT disc cells from their stalks. Material was then drained through a series of sieves (220 μm, 120 μm, and 25 μm). GT disc cells were collected from the 25 μm sieve and resuspended in ice-cold mannitol buffer (0.2 M mannitol, 0.05 M Tris-HCl, 0.005 M MgCl_2_, 0.01 M KCl, 0.0005 M K_2_HPO_4_, 0.001 M EGTA; pH 6.0) in a 50 mL tube. The washing and trichome disc cell collection were repeated twice more on the same batch of flowers. Once collection was completed, the trichome disc cells were gently washed three times with fresh mannitol buffer and then resuspended in a new 50 mL tube to a final volume of 30 mL (Supplementary Figure S7).

### 5.3. In Vitro Phytohormone Treatment

Sterile basal agar plates [1.5% (*w*/*v*) agar] supplemented with 0.02 M sucrose and 0.02 M raffinose were prepared with different phytohormonal treatments in order to test the response of isolated trichome disc cells under *in vitro* conditions. The hormone concentrations applied were as follows: 20 μM (+)-cis,trans-abscisic acid (Astral Scientific, Taren Point, NSW, Australia; A-1003); 20 μM salicylic acid (Sigma-Aldrich, St. Louis, MO, USA; S7401); 20 μM (±) jasmonic acid (Sigma-Aldrich, J2500); 1 mM sodium diethyldithiocarbamate (Sigma-Aldrich, 228680); 20 μM brassinolide (Sapphire Bioscience, Redfern NSW, Australia; 21594); 20 μM ethephon (Sigma-Aldrich, C0143); 20 μM gibberellic acid (ChemSupply, Gillman, SA, Australia; GL003); 20 μM indole acetic acid (Biosynth, Staad, Switzerland; FI09866); 20 μM kinetin (Acros Organics, Antwerp, Belgium; 226500050); start point control (CTR_0h); end point control (CTR_24h). Phytohormones were prepared as 1000X stock solutions dissolved in 100% (*v*/*v*) ethanol and then added to the molten agar post-sterilisation. Equivalent amounts of 100% (*v*/*v*) ethanol were added to molten control agar post-sterilisation as the treatments. While working under sterile conditions in a laminar flow hood, the trichome disc suspension (500 μL) in mannitol buffer (0.2 M mannitol, 0.05 M Tris-HCl, 0.005 M MgCl_2_, 0.01 M KCl, 0.0005 M K_2_HPO_4_, 0.001 M EGTA; pH 6.0) was pipetted onto the petri plates, which were then gently tilted to spread the cells across before air drying and sealing with parafilm.

The plates were incubated in an environmental chamber (Sanyo, Osaka, Japan; MLR-350H) set to 26 °C with 24 h light (200 µmol m^2^ s^−1^) for a period of 25.5 h. Continuous light was used to maintain GT disc cells in a state where cannabinoid anabolism is most active [33]. Each plate was considered a biological replicate, with four replicates prepared per treatment (*n* = 4). After incubation, trichome disc cells were harvested from each plate using a cell scraper, resuspended in 900 μL of mannitol buffer and then split into aliquots. Trichome disc cells were spun down at 4 °C 12,000× *g* for 2 min to remove the supernatant before flash freezing in liquid nitrogen and storage at −80 °C.

### 5.4. Protein Extraction

Protein was extracted from four biological replicates (*n* = 4) per treatment following methods as described in Dimopoulos et al. [33]. Pelleted trichome cells were first disrupted by adding a 5 mm steel ball into the sample tube and shaken while frozen at 26 Hz for 20 s on a TissueLyser II (Qiagen, Hilden Germany). While working on ice, the powdered pellets were then each resuspended with 220 μL of suspension buffer (0.4 M mannitol, 1.086 M glycerol, 0.0054 M Tris-MES, 0.0013 M dithiothritol) and vortexed. A 100 μL aliquot of the suspension was frozen away at −80 °C, and a second one was taken for protein extraction, where 50 μL of each 10X TE buffer, 0.5% (*w*/*v*) sodium deoxycholate, and 72% (*w*/*v*) trichloroacetic acid were added sequentially to the sample and vortexed with each addition. Protein extraction samples were incubated on ice for one hour and then centrifuged at 14,000× *g* and 4 °C for 20 min. The supernatant was then carefully aspirated without disturbing the protein pellets, and pellets were resuspended in 100 μL 90% (*v*/*v*) methanol before incubating overnight at −20 °C.

The next day, protein samples were centrifuged at 14,000× *g* and 4 °C for 20 min. The supernatant was aspirated, and the protein pellet was resuspended in 100 μL of ice cold 90% (*v*/*v*) methanol. This pellet washing was repeated three times, after which the pellets were centrifuged and the supernatant was aspirated for a final time. The protein pellets were then left to dry in a fume hood for approximately one hour, with the open lids covered with a Kimwipe. Once dry, the pellets were stored at −80 °C prior to mass spectrometry analysis by Sequential Window of All Theoretical Mass Spectra (SWATH-MS) at the IMB Proteomics facility of the University of Queensland.

### 5.5. LC–MS/MS Analysis of Cannabis GT Proteome

Proteomic analysis of trichome-derived peptides was as described in Dimopoulos et al. [33] and conducted on an Ekspert nano LC400 uHPLC system (SCIEX, Framingham, MA, USA), interfaced with a TripleTOF 6600 QTOF mass spectrometer (SCIEX, Canada), which was equipped with a PicoView nanospray source (New Objective, Littleton, MA, USA). Separation of peptides was facilitated by a dual-column setup, employing a 5 mm × 300 mm C18 3 µm trap column (SGE Analytical Science, Ringwood, VIC, Australia) and a 75 µm × 150 mm ChromXP C18 CL 3 µm analytical column (SCIEX, Canada). The elution gradient was formulated with 0.1% formic acid in both water (Solvent A) and acetonitrile (Solvent B), increasing Solvent B from 2% to 40% over 60 min, followed by a ramp to 90% for 5 min, and maintained at 90% for another 5 min before re-equilibrating to 2%. The flow rate of the mobile phase was maintained at 400 nL/min, and the column was thermostatically controlled at 45 °C. Sample peptides, in 5 µL aliquots, were introduced to the trap column at a flow rate of 10 µL/min for 5 min, then transferred to the analytical column at a consistent flow rate. The SWATH acquisition mode was employed for MS/MS analysis, capturing 100 product ion spectra per cycle across the range of *m*/*z* 350 to 1500, with each product ion window defined at 6 Da. Variable collision energies were applied, ranging from 16 to 60 V, with an energy spread of 5 V. The TOF-MS scans were conducted with an acquisition time of 50 ms, and each product ion scan was allotted 25 ms.

Data collection and processing were executed using Analyst TF software version 1.7 (SCIEX). The SWATH-MS data underwent spectral alignment and targeted data extraction using the SWATH Processing Micro App within PeakView software (Version 1.2, SCIEX), leveraging an inhouse assembled spectral library built from Hindu Kush spectral data [33]. The extraction parameters were set with a 15 min window and included ten peptides per protein, five transitions per peptide, a peptide confidence threshold of greater than 95%, exclusion of shared peptides, and an extracted ion chromatogram width of 75 ppm.

### 5.6. Metabolite Extraction from Cannabis GT

Cannabinoids were extracted from GTs with one aliquot taken for each biological replicate. While still frozen, a 5 mm steel ball was added to each tube and cells were pulverised by shaking on a TissueLyser II (Qiagen) at 20 Hz for 12 s. Once pulverised, 1000 μL of 100% (*v*/*v*) ethanol (HPLC-grade) was added to each sample, the mixture was then briefly vortexed, and then the liquid sample was transferred to a new pre-weighed 2 mL tube. The samples were then sonicated (Soniclean^®^, Dudley Park, SA, Australia; Digital benchtop ultrasonic cleaner) for 30 min at 43 kHz ± 2 kHz sweep bandwidth with 20 Hz pulses and centrifuged at 16,000× *g* for 20 min at 20 °C, and then the supernatant was transferred to a new tube and stored at −20 °C for further analysis. The pellet was left to air-dry in a fume hood overnight, and the pellet dry weight was measured. On day of analysis, the stored supernatant was sonicated once more for 30 min at 30 Hz and then centrifuged at 16,000× *g* for 5 min to pellet any undissolved precipitate with supernatant transferred to an HPLC vial for analysis.

### 5.7. HPLC-UV Analysis of Cannabinoids from Cannabis GT Cells

Targeted cannabinoid analysis was performed using HPLC (Agilent Technologies, Santa Clara, CA, USA; LC 1260 Infinity II) with a UV lamp and an Agilent 1260 Infinity II Diode Array Detector HS (Agilent Technologies) using a method adapted from Hewavitharana et al. [83]. This method allows for the quantification of CBGA, THCA, CBDA, CBCA, cannabidivarinic acid (CBDVA), tetrahydrocannabivarinic acid (THCVA), and tetrahydrocannabinol (THC). Separation was attained by using a reverse phase column (Agilent Technologies; Agilent Infinity Poroshell 120, HPH-C18, 2.1 × 150 mm, 2.7 µm, narrow bore LC column) with a water/acetonitrile/methanol gradient. A 1 µL aliquot was injected for each sample. The column temperature was set at 30 °C with a flow rate of 0.3 mL/min. The mobile phase first started with water/acetonitrile/methanol (35/50/15, *v*/*v*/*v*) and changed to water/acetonitrile/methanol (1/99/0, *v*/*v*/*v*) over a period of 10 min and was held for 2 min and then changed back to water/acetonitrile/methanol (35/50/15, *v*/*v*/*v*) over a period of 1 min and then held for 5 min. Chromatographic peaks were analysed using Agilent OpenLab CDS (version 2.7), with CBGA and THCA identified by retention time, with their absorbance signals measured at 270 nm and calibrated against commercially available cannabinoid standards (Novachem, Heidelberg West, VIC, Australia). Cannabinoid content was expressed as mg g^−1^ (DW) GT disc cells.

### 5.8. Statistical and Bioinformatic Analyses

Statistical analyses were conducted in R (v4.3.1) using various packages. Two-sample Student’s *t*-test was used to determine significant differences for protein and metabolite abundance, between treatments and control. A *p*-value < 0.05 with either a fold-change < 0.67 or a fold-change > 1.5 was considered significant for proteins, and a *p*-value < 0.05 was considered significant for metabolites. Principal component analysis (factoextra package v1.0.7) was used to cluster GT proteome samples. Heatmaps were created using the pheatmap (v1.0.12) and were hierarchically clustered by Ward clustering using Euclidean distance. Like in Dimopoulos et al. [33] and other studies with non-model plants [84], the functional annotation of the identified cannabis GT proteins was determined by aligning sequences to the best BLASTP matches within the National Center for Biotechnology Information (NCBI) and UniprotKB/SwissProt databases, utilising arabidopsis as a reference organism. The threshold for significance was set at an e-value of less than 1.0 × 10^−5^. Gene ontology enrichment analysis of groups of differentially abundant proteins was conducted using the clusterProfiler (v4.12.6) package [85,86] with the R package org.At.tair.db: genome-wide annotation package for arabidopsis (v3.19.1). Enriched ontology terms were considered significant when the adjusted (Benjamini–Hochberg) *p*-value < 0.05. Gene ontology semantic similarity analysis was performed with the GOSemSim (v2.30.2) package for R [87].

## Data Availability

The primary data supporting this study include the raw mass spectrometry proteomics data and metabolite data. All primary data have been deposited (ID 991013205013802368) to the Southern Cross University research portal (https://researchportal.scu.edu.au/) and are openly available (19 December 2024) at https://doi.org/10.25918/data.382.

## Supplementary Materials

The following supporting information can be downloaded at https://www.mdpi.com/article/10.3390/plants14050694/s1. Supplementary Dataset S1—Statistics results for glandular trichome proteome analysis. Supplementary Figure S1—Enriched GO terms of global increased and decreased DAPs. Supplementary Figure S2—Enriched GO terms and proteins affected by ethephon. Supplementary Figure S3—Abundances of key proteins in controls at 0 h and 24 h. Supplementary Figure S4—Photos of control time course trial. Supplementary Figure S5—Abundances of phytohormone regulatory proteins. Supplementary Figure S6—Abundances of photosystem proteins. Supplementary Figure S7—Photos of high purity trichome disc cells. Supplementary Figure S8—Photos comparing disc cells with and without storage cavities.
