# Supplementary material for: An In Vitro Phytohormone Survey Reveals Concerted Regulation of the Cannabis Glandular Trichome Disc Cell Proteome
Source: Plants (Basel). 2025 Feb 24;14(5):694. doi: 10.3390/plants14050694 (PMC11901956; doi:10.3390/plants14050694)
Supplement: Supplementary file 1 [file plants-14-00694-s001.zip › plants-3457515-Supplementary Figures S1-S8.pdf]

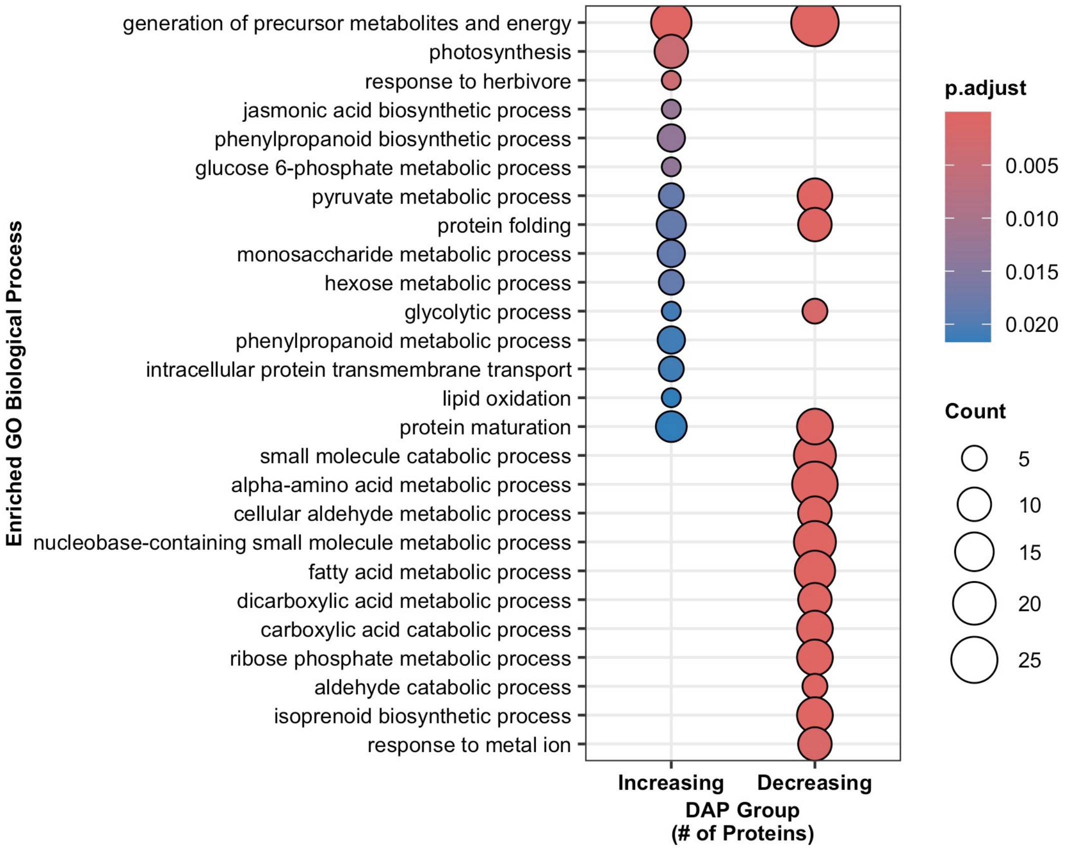

Supplementary Figure S1. *In vitro* agar plate incubation of live isolated *Cannabis* glandular trichome disc cells under different phytohormone conditions for 24 hours (n=4) that include 0h control (CTR_0h), 24h control (CTR_24h), abscisic acid (ABA), brassinolide (BRA), diethyldithiocarbamate (DIE), ethephon (ETH), gibberellic acid (GA3), kinetin (KIN), jasmonic acid (JAS), and salicylic acid (SAL). Of 1400 quantified proteins, 448 were differentially abundant proteins (DAPs) that showed significance ($p < 0.05$ and fold-change either < 0.67 or > 1.5) in any of the phytohormone treatments. Gene ontology (GO) biological process enrichment analysis was conducted on the DAPs that grouped in either the increasing or decreasing fold-change hierarchical clusters to reveal the top pathways affected by phytohormone treatments. Count represent the number of proteins associated with the specific biological process.

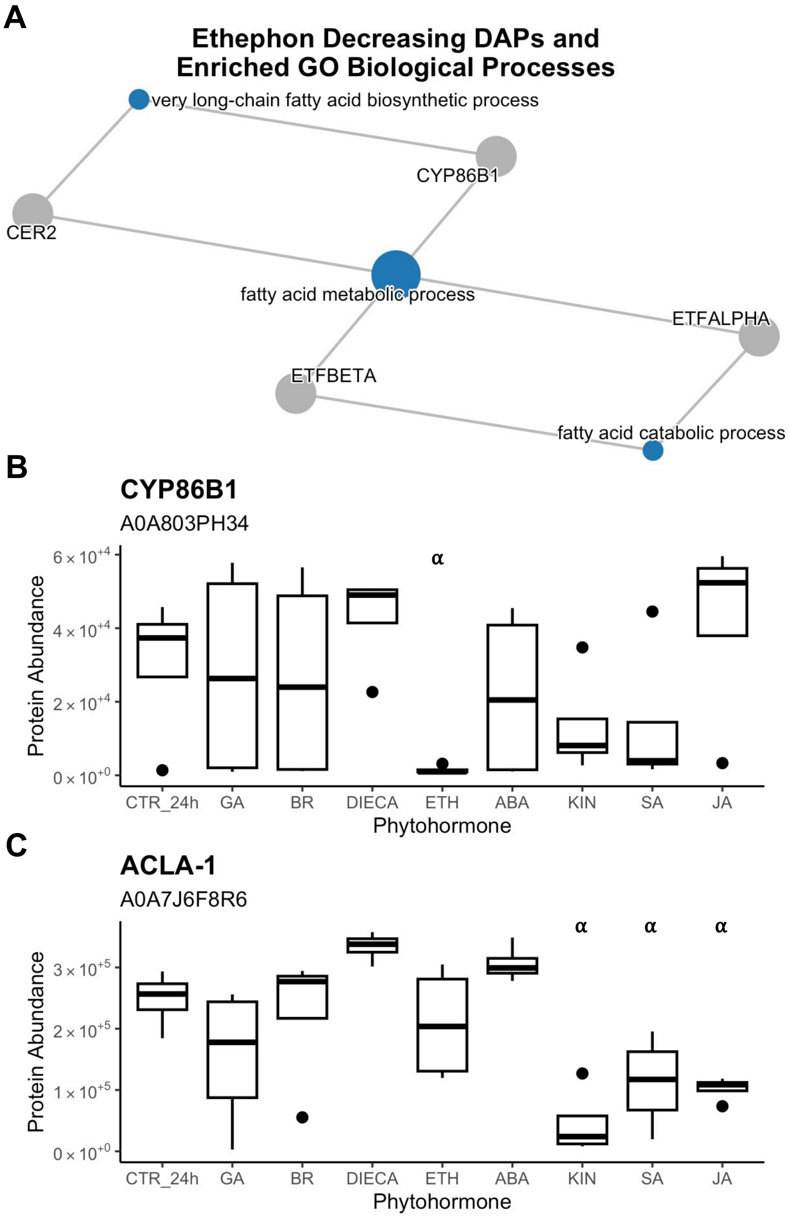

Supplementary Figure S2. Gene ontology enrichment analysis map of decreasing differentially abundant proteins when *Cannabis* glandular trichomes were treated with ethephon, blue dots represent biological processes and grey dots represent proteins **(A)**. Abundance of proteins (n=4) of interest **(B-C)** with significant difference (denoted by **α**) in comparison to control determined by two-sample student's t-test (p-value<0.05 and when fold-change <0.67 or >1.5). Abbreviations: ACLA-1, ATP citrate lyase A-1; ; CTR_24h, 24 h control; ABA, abscisic acid; BR, brassinolide; DIECA, diethyldithiocarbamate; ETH, ethephon; GA, gibberellic acid; KIN, kinetin; JA, jasmonic acid; SA, salicylic acid. *Cannabis* UniProt accession number listed underneath protein names.

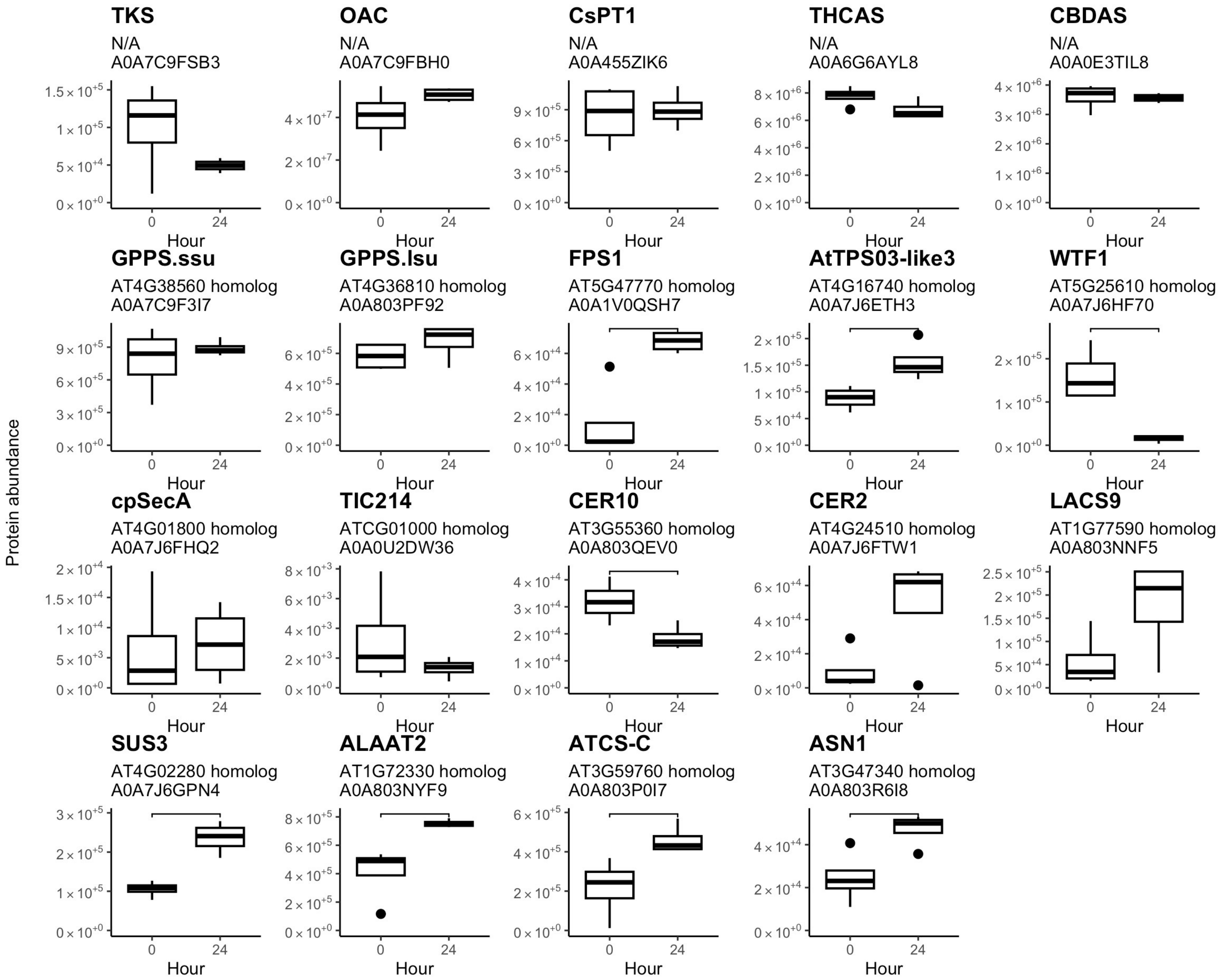

Supplementary Figure S3. Abundance of key proteins in isolated *Cannabis* glandular trichome disc cells control samples at 0h and 24h timepoints during *in vitro* incubation (n=4). Boxplots represent median, interquartile range, maximum, minimum and outliers (closed circles). Significance (denoted by horizontal bracket) determined by two sample student’s t-test (p-value <0.05) by comparing 24h and 0h timepoints.

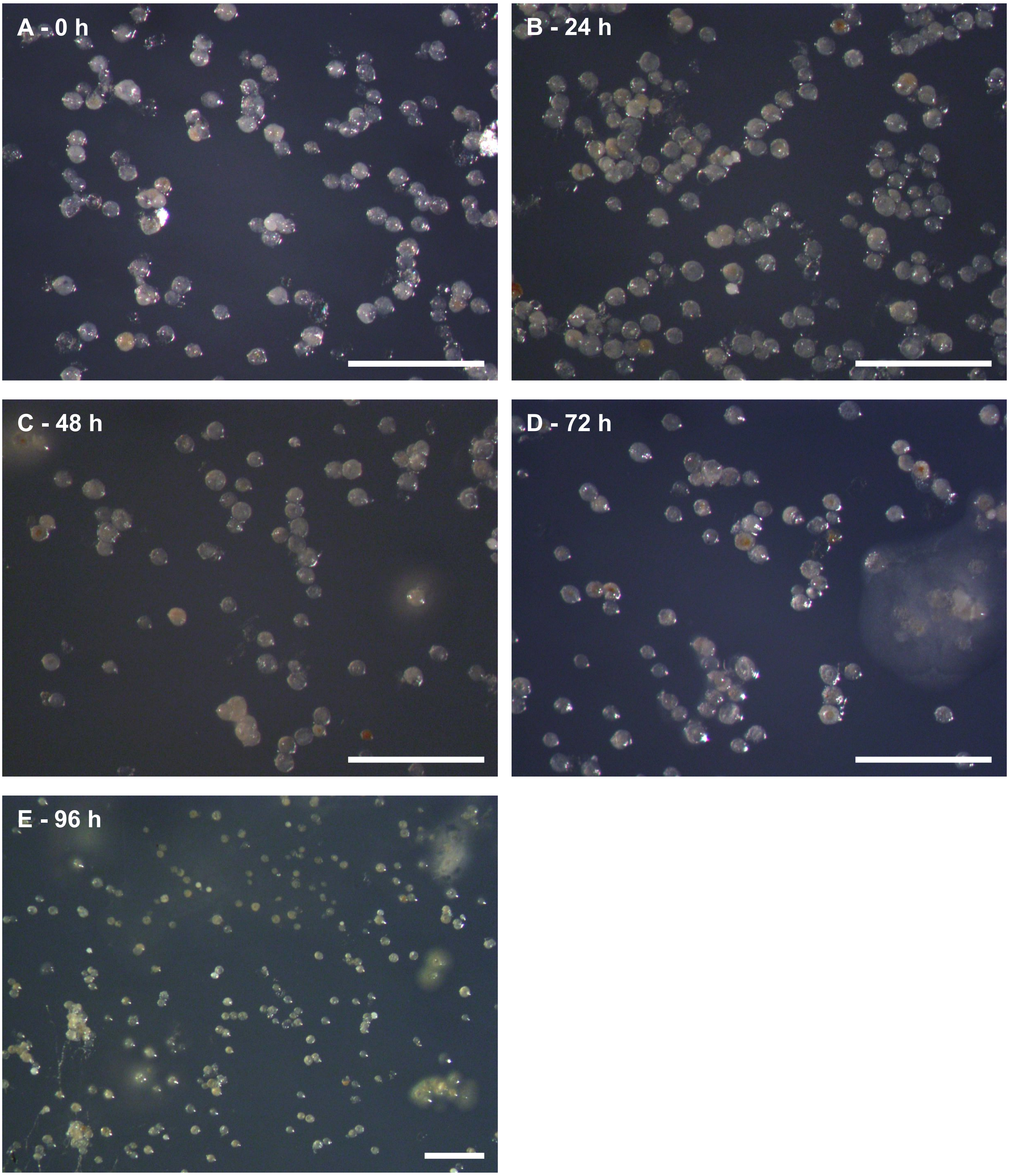

Supplementary Figure S4. Light microscope images of *Cannabis* glandular trichomes on the surface of basal media plates as part of the *in vitro* assay under control conditions during a time course trial. Individual plates were prepared, incubated and then imaged at discrete timepoints ranging from 0h to 96 h **(A - E)** for any observable growth of fungal or bacterial contaminants. Scale bars represent 1 mm. Photo credit: Nicolas Dimopoulos.

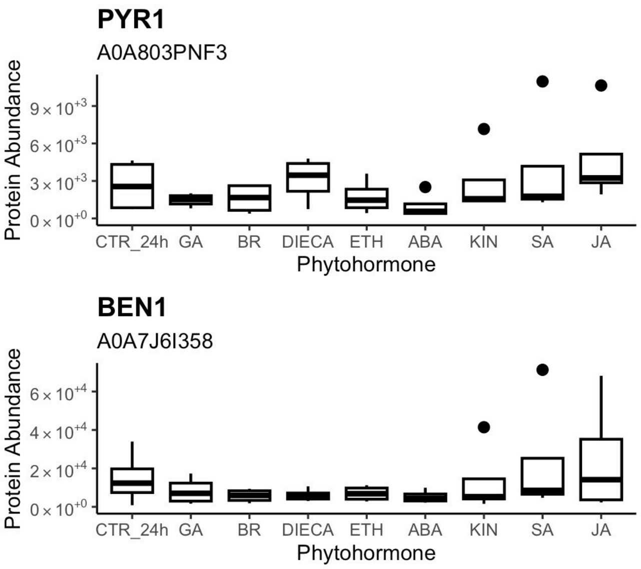

Supplementary Figure S5. Abundance of regulatory phytohormone signalling proteins (n=4) with significant difference (denoted by **α**) in comparison to control determined by two-sample student's t-test (p-value<0.05 and when fold-change <0.67 or >1.5). Abbreviations: ACLA-1, ATP citrate lyase A-1; ; CTR_24h, 24 h control; ABA, abscisic acid; BR, brassinolide; DIECA, diethyldithiocarbamate; ETH, ethephon; GA, gibberellic acid; KIN, kinetin; JA, jasmonic acid; SA, salicylic acid. Protein names and *Cannabis* UniProt accession number listed underneath protein names

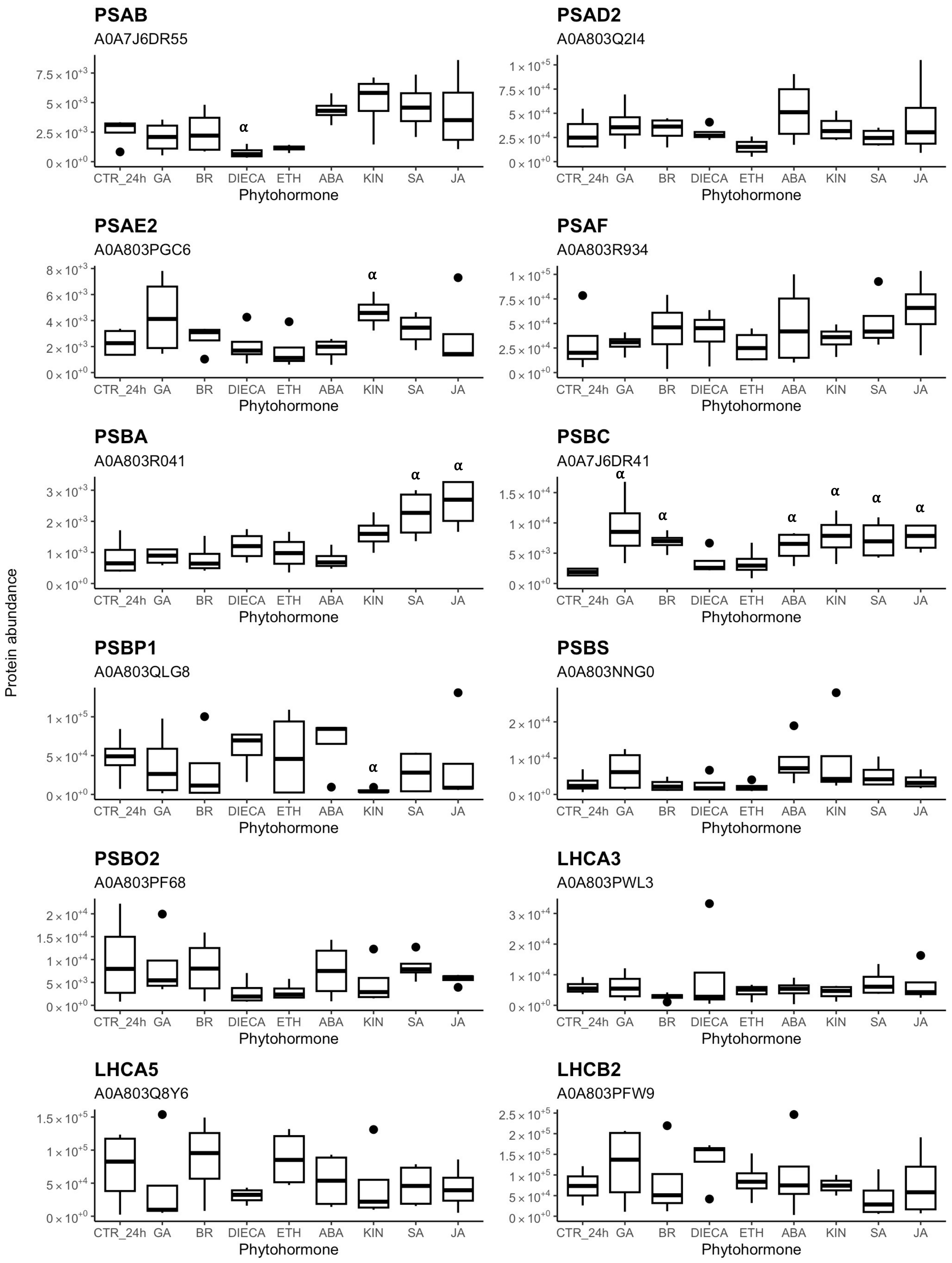

Supplementary Figure S6. Abundance of photosystem proteins (n=4) with significant difference (denoted by **α**) in comparison to control determined by two-sample student's t-test (p-value<0.05 and when fold-change <0.67 or >1.5). Abbreviations: ACLA-1, ATP citrate lyase A-1; ; CTR_24h, 24 h control; ABA, abscisic acid; BR, brassinolide; DIECA, diethyldithiocarbamate; ETH, ethephon; GA, gibberellic acid; KIN, kinetin; JA, jasmonic acid; SA, salicylic acid. Protein names and *Cannabis* UniProt accession number listed underneath protein names

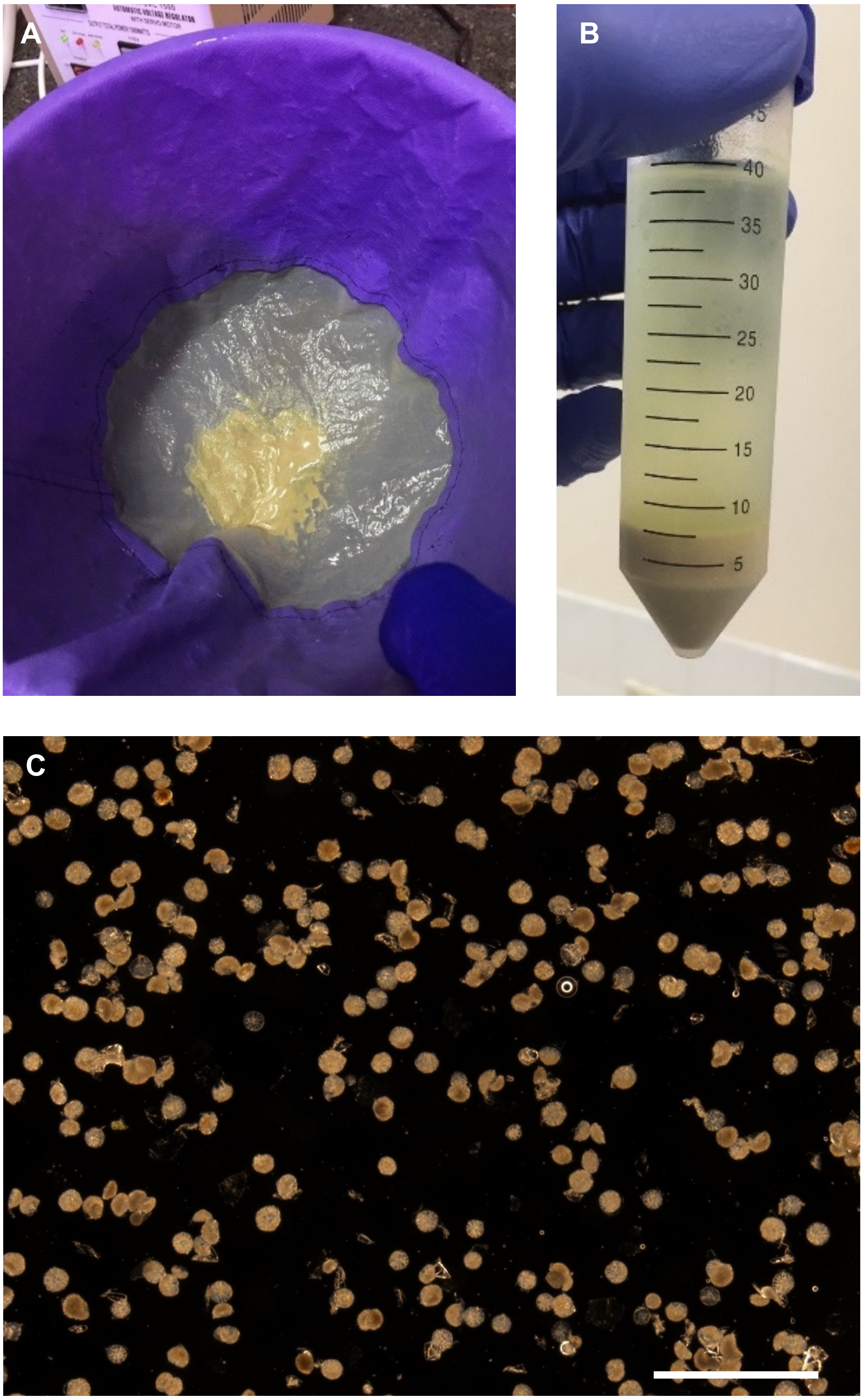

Supplementary Figure S7. The cannabis glandular trichome (GT) isolation method results in a pure collection of live GTs. The GTs are first collected from the sieve from which they are captured **(A)**, afterwards suspended in mannitol buffer **(B)**, and finally checked for purity under a microscope before used for the *in vitro* assay **(C)**. Scale bar represents 1mm. Photo credit: Nicolas Dimopoulos.

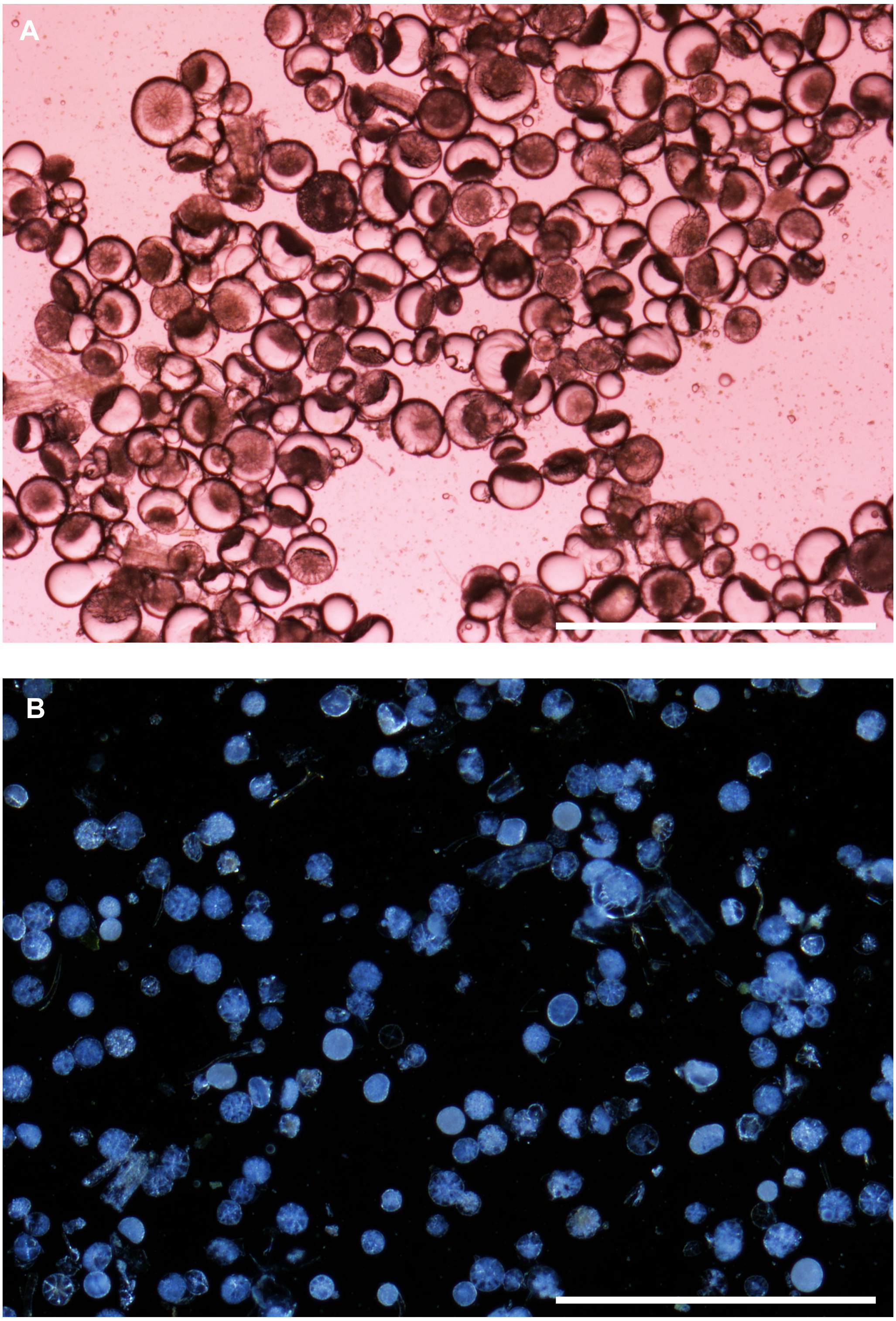

Supplementary Figure S8. Examples of cannabis glandular trichomes when isolated with their storage cavity kept intact **(A)** and when the storage cavity was removed **(B)**.
